# Gene Expression in Skeletal Muscle Biopsies from People with Type 2 Diabetes and Relatives: Differential Regulation of Insulin Signaling Pathways

**DOI:** 10.1371/journal.pone.0006575

**Published:** 2009-08-11

**Authors:** Jane Palsgaard, Charlotte Brøns, Martin Friedrichsen, Helena Dominguez, Maja Jensen, Heidi Storgaard, Camilla Spohr, Christian Torp-Pedersen, Rehannah Borup, Pierre De Meyts, Allan Vaag

**Affiliations:** 1 Receptor Systems Biology Laboratory, Hagedorn Research Institute, Novo Nordisk, Gentofte, Denmark; 2 Steno Diabetes Center, Gentofte, Denmark; 3 Department of Clinical Biochemistry, Rigshospitalet, University of Copenhagen, Copenhagen, Denmark; Texas Tech University Health Sciences Center, United States of America

## Abstract

**Background:**

Gene expression alterations have previously been associated with type 2 diabetes, however whether these changes are primary causes or secondary effects of type 2 diabetes is not known. As healthy first degree relatives of people with type 2 diabetes have an increased risk of developing type 2 diabetes, they provide a good model in the search for primary causes of the disease.

**Methods/Principal Findings:**

We determined gene expression profiles in skeletal muscle biopsies from Caucasian males with type 2 diabetes, healthy first degree relatives, and healthy controls. Gene expression was measured using Affymetrix Human Genome U133 Plus 2.0 Arrays covering the entire human genome. These arrays have not previously been used for this type of study. We show for the first time that genes involved in insulin signaling are significantly upregulated in first degree relatives and significantly downregulated in people with type 2 diabetes. On the individual gene level, 11 genes showed altered expression levels in first degree relatives compared to controls, among others *KIF1B* and *GDF8* (myostatin). *LDHB* was found to have a decreased expression in both groups compared to controls.

**Conclusions/Significance:**

We hypothesize that increased expression of insulin signaling molecules in first degree relatives of people with type 2 diabetes, work in concert with increased levels of insulin as a compensatory mechanism, counter-acting otherwise reduced insulin signaling activity, protecting these individuals from severe insulin resistance. This compensation is lost in people with type 2 diabetes where expression of insulin signaling molecules is reduced.

## Introduction

Type 2 diabetes is a complex and multi-factorial disease involving both genetics and pre- and postnatal environmental etiological factors. The genetic importance in the pathogenesis of type 2 diabetes is indicated by several lines of evidence from studies of both twins and first degree relatives of people with type 2 diabetes [1]. Additionally, type 2 diabetes segregates in families, and there are substantial differences in the prevalence between ethnic groups and races [1]. Finally, insulin resistance is maintained in skeletal muscle cell cultures started from biopsies taken from people with type 2 diabetes and insulin resistant individuals signifying that it is not only the surrounding milieu that causes the molecular defects [2]–[4].

The underlying genetics of type 2 diabetes is very complex and it is clear that several genes play a role making this a polygenic disease. Furthermore, there are several different combinations of the so-called ‘diabetogenes’ that can lead to type 2 diabetes under the influence of certain environmental conditions [5]–[7]. Several new type 2 diabetes gene regions have recently been identified [8], [9], [10]. Whether these SNPs in or close to specific genes are part of the underlying pathogenesis or simply markers of the disease is still not known, although some of these variants have been linked to impaired β-cell function and insulin secretion [11].

Skeletal muscle accounts for approximately 75% of the glucose uptake after a meal, and accordingly has a major impact on overall glucose homeostasis [12]. It has previously been shown that skeletal muscle from Mexican Americans and Europeans with type 2 diabetes has an altered gene expression profile compared to healthy control individuals [13]–[15]. These changes can either be a secondary effect of a changed metabolic milieu, a direct consequence of reduced insulin signaling, or be part of the primary cause of the disease.

First degree relatives of people with type 2 diabetes are as a group very interesting since they have a greatly increased risk of developing type 2 diabetes compared to the background population [16]. In relatives that have not developed insulin resistance, changes in gene expression are not secondary to an altered metabolic milieu since these individuals are not subjected to any metabolic dys-regulation or decreased level of insulin action [17].

Given the fact that type 2 diabetes is a polygenic disorder, the microarray technology simultaneously measuring the expression of thousands of genes is well suited for studies of this disease. We determined the expression profiles in skeletal muscle from people with type 2 diabetes, first degree relatives, and healthy control individuals by microarray experiments. All subjects were Caucasian males and biopsies were taken after a controlled metabolic period of a two hour hyperinsulinemic euglycemic clamp. Our results show for the first time that insulin signaling is significantly downregulated in people with type 2 diabetes, whereas it is significantly upregulated in first degree relatives. Furthermore, we identify several new genes in skeletal muscle from first degree relatives that have an altered gene expression compared to healthy controls.

## Methods

### Clinical characterization of subjects and biopsy procedure

Male subjects comprised three experimental groups; healthy controls, people with type 2 diabetes, and first degree relatives (Table 1). The first degree relatives were defined as people with at least 50% of their genes in common with a person with type 2 diabetes, and were not related to the type 2 diabetic patients participating in this study. In vivo insulin action was measured as M-values (mg glucose/kg FFM/min) as determined during a 2 hour 40 mU/m^2^/min hyperinsulinemic euglycemic clamp. The insulin concentration was acutely raised and maintained by a continuous infusion of insulin and the glucose concentration was held constant at basal levels (5 mmol/L), by variable glucose infusion. After 2 hours, biopsies were taken from the vastus lateralis muscle of each subject using a Bergström needle under local anesthesia. Samples were immediately frozen in liquid nitrogen and saved for later use.

**Table 1:** Clinical characteristics of subjects.

		C n = 15		D n = 5		R n = 15			p-value C vs. D	p-value C vs. R
		Mean	SD	Mean	SD	Mean	SD			
Age (yr)		44.6	11.5	52.2	8.8	43.5		8.0		
Height (m)		1.83	0.07	1.80	0.03	1.79		0.08		
Weight (kg)		90.8	13.4	110.0	17.8	91.8		11.4	0.07	
W/H ratio		0.92	0.06	1.07	0.04	0.93		0.03	<0.001	
BMI (kg/m^2^)		27.20	3.97	33.98	4.52	28.50		3.07	0.02	
FFM (kg)		68.70	6.66	76.32	7.58	70.90		6.66	0.05	
Systolic BP (mm Hg)		124	13	141	12	128		12	<0.001	
Diastolic BP (mm Hg)		73	11	85	8	78		12	0.03	
FFA (µmol/L)		266	137	530	206	267		113	0.01	
F-p Cholesterol (mmol/L)		5.22	1.01	4.82	0.89	5.71		0.96	<0.001	
F-p HDL (mmol/L)		1.32	0.39	1.02	0.18	1.17		0.31	0.04	
F-p Triglyceride (mmol/L)		1.45	0.79	3.80	5.47	1.85		0.90		
F-p LDL (mmol/L)		3.25	0.90	2.9	0.83	3.69		0.93		
F-p VLDL (mmol/L)		1.03	1.42	0.63	0.22	0.82		0.42		
Blood Glucose (mmol/L)	Basal	4.81	0.70	7.30	1.15	4.88		0.71	0.01	
Blood Glucose(mmol/L)	Insulin	4.87	0.27	4.86	0.15	4.85		0.34		
Plasma Insulin (pmol/L)	Basal	39.3	36.2	63.4	13.4	65.9		36.8	0.04	0.06
Plasma Insulin (pmol/L)	Insulin	384	97	466	26	549		248	0.01	0.03
Plasma C-peptide (pmol/L)	Basal	607	310	804	239	745		296		
GOX (mg glu/kg FFM/min)	Insulin	3.35	0.33	1.90	0.18	3.45		0.34	<0.001	
FOX (mg glu/kg FFM/min)	Insulin	0.10	0.01	1.04	0.10	0.13		0.26	<0.001	
M-value (mg glu/kg FFM/min)	Insulin	11.40	3.75	5.91	1.42	9.21		3.73	0.01	
NOGM (mg glu/kg FFM/min)	Insulin	7.65	3.39	4.01	1.47	6.00		3.53	0.04	

Average clinical data for all subjects in the three different experimental groups: healthy controls (C), people with type 2 diabetes (D), and first degree relatives (R). The control and relative groups consisted of 15 individuals each, whereas the type 2 diabetes group consisted of 5 individuals. All subjects were Caucasian males. Glucose and lipid oxidation was calculated using the equations suggested by Frayn [36] NOGM was calculated as the M-value – glucose oxidation rate. P-values are listed when significant. SD: standard deviation, W/H: waist/hip, FFM: fat-free mass, BP: blood pressure, FFA: free fatty acids, HDL: high density lipoprotein, LDL: low density lipoprotein, VLDL: very low density lipoprotein, GOX: glucose oxidation, FOX: fat oxidation, NOGM: non-oxidative glucose metabolism, F-p: fasting plasma.

The study protocol was in accordance with the Helsinki Declaration II, and approved by The Danish Research Agency (KA 01122 g), and by The Danish Data Protection Agency (J.nr. 2001-41-1531). All subjects signed an informed consent form before entering the study.

Statistical analyses were performed with SAS Statistical Analysis Package (SAS Institute, Cary, NC, version 8.2). Two-sided Student's *t*-test was used to identify statistically significant differences between the groups. Data are presented as mean values±SD, and values of *P*≤0.05 were considered to be significant.

### RNA isolation, cRNA production and fragmentation, array hybridization and scanning

After homogenization, total RNA was isolated from the skeletal muscle biopsies using Trizol reagent from Invitrogen as specified by the manufacturer. The RNA subsequently went through a clean-up step using the RNeasy Mikro kit from Qiagen. Fragmented biotinylated cRNA was made and hybridized to Affymetrix Human Genome U133 Plus 2.0 Arrays and scanned following guidelines from Affymetrix (www.affymetrix.com). These arrays contain approximately 54,000 probesets representing approximately 47,000 transcripts.

### Data analysis

Cell intensity files (CEL files) were generated in the program GCOS from Affymetrix. A quality control report was subsequently made using Bioconductor, and the data were modeled using the RMA (Robust Multichip Average) approach [18], [19]. Comparisons of individual genes between groups were made in dChip (http://biosun1.harvard.edu/complab/dchip/). The fold change (FC) was set to >1.2, the p-value<0.05 (unpaired t-test), with a lower 90% confidence bound of FC, and the difference between experiment and control intensity value was set to be more than 30. The false discovery rate (FDR) was determined using a permutation approach and should be less than 5%.

Functional analyses were made using the program GenMAPP/MAPPFinder [20] (http://www.genmapp.org/). Here the criteria were set to: FC>1.2, p-value <0.05, and the intensity value >30. Functional analyses were also performed using the program Ingenuity Pathway Analysis (IPA), using the FC>1.2, p-value <0.05 criteria. The microarray data is described in accordance with MIAME guidelines.

### Quantitative RT-PCR

Quantitative RT-PCR was performed for selected genes in order to validate the results obtained in the microarray study. cDNA was produced from 0.5 µg of each RNA sample using the ‘High Capacity cDNA Reverse Transcription Kit’ from Applied Biosystems. The last step of the experiments was performed using TaqMan Low Density Arrays (customized) and the ‘TaqMan Universal PCR Master Mix’ both from Applied Biosystems following company guidelines. The arrays were run on the 7900HT system and data were analyzed using the SDS 2.1 software from Applied Biosystems. The Ct value for each sample was determined at least twice on different arrays, and the average was used to calculate relative fold changes (FC = 2^−ΔΔCt^). The PPIA (cyclophilin A) gene was used as an endogenous control. Calculating the FC in this way, only one value including all replicates is obtained and accordingly standard deviations are reported for Ct values and not fold changes.

### Western blot protein assessment of the Insulin Receptor and PGC1α

Protein lysates (20 µg of total protein) from the same skeletal muscle samples used for the microarray study were separated on 10% BIS-TRIS gels and proteins were transferred to nitrocellulose membranes (all from Invitrogen). After blocking, the membranes were incubated overnight with primary antibodies against IRbeta (sc-711, Santa Cruz Biotechnology) and PGC-1alpha (sc-5816, Santa Cruz Biotechnology) followed by a second incubation with HRP-conjugated anti-rabbit antibody from Cell Signaling (#7074). The signal was detected with LumiGLO reagent (#7003, Cell Signaling) and bands were visualized using the LAS-3000 Image-reader from Fujifilm. Band intensities were quantified using the Multi Gauge V2.0 software (Fujifilm).

## Results

We determined the gene expression profiles in skeletal muscle biopsies from healthy individuals, people with type 2 diabetes, and first degree relatives. For simplicity reasons these groups will be termed ‘C’ (controls), ‘D’ (diabetics), and ‘R’ (relatives). Gene expression values were determined using the microarray technology from Affymetrix as described above. All subjects were Danish Caucasian males, and all biopsies were taken after a 2 hour hyperinsulinemic euglycemic clamp as previously described [21]. Clinical characteristics determined for the different experimental groups are listed in Table 1. The ‘C’ and the ‘R’ group consisted of 15 individuals each, whereas the ‘D’ group consisted of 5 individuals. The ‘D’ group was slightly older and significantly more obese as compared to the two other groups. Additionally, they were hyperglycemic, hyperinsulinemic, had increased free fatty acid (FFA) levels and increased blood pressure compared to healthy controls. The first degree relatives were healthy, normoglycemic and mildly insulin resistant as revealed by their M-values. However, they were notably hyperinsulinemic compared to the controls.

### Genes differentially expressed in skeletal muscle from people with type 2 diabetes or first degree relatives

The expression levels of individual genes were compared between groups using the program dChip. All genes found to be regulated and their fold changes are listed in the online Table S1. The genes mentioned in either the Results or the Discussion section are listed in Table 2 and Table 3.

**Figure 1:**
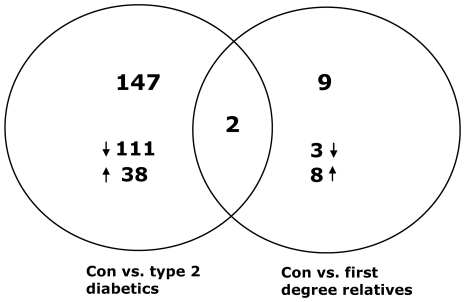
Number of differentially expressed genes. Diagram showing the number of genes found to be differentially expressed in skeletal muscle biopsies from people with type 2 diabetes and first degree relatives compared to healthy controls in a dChip analysis of the generated microarray data. Criteria used in the analysis were set as described in the Methods section. The number of up- and downregulated genes in each group is indicated. Only 2 genes were found to have an altered expression in both groups, namely *TncRNA* and *LDHB*. LDHB is a key-enzyme in anaerobic glycolysis and had a reduced expression in both the ‘R’ and the ‘D’ group compared to controls.

Employing the cutoffs described in the Methods section, 149 genes were found to be differentially expressed in the ‘D’ group compared to controls. The majority of these genes were downregulated (Figure 1). The generated genelist included several noteworthy genes like the insulin receptor (*INSR*), insulin receptor substrate 2 (*IRS2*), protein phosphatase 1 (*PPP1CB*), lipoprotein lipase (*LPL*), hexokinase 2 (*HK2*), phosphorylase kinase (*PHKA1*), forkhead box O3A (*FOXO3A*), histone deacetylase 7A (*HDAC7A*), and NADH dehydrogenase (*NDUFS1*) (Table 2).

**Table 2:** Genes differentially expressed in people with type 2 diabetes.

Gene symbol	Gene name	FC microarray	qRT-PCR	DChip criteria
*Insulin Signaling*				
INSR	Insulin receptor	−1.66	Yes	Yes
IGF1R	Insulin-like growth factor 1 receptor	−1.37	Yes	
IRS2	Insulin receptor substrate 2	−1.58	Yes	Yes
PIK3CA	Phosphoinositide-3-kinase, catalytic, alpha	−1.32	Yes	
PIK3CD	Phosphoinositide-3-kinase, catalytic, delta	−1.44		
PIK3R1	Phosphoinositide-3-kinase, regulatory subunit 1	−1.49		
PDPK1	3-phosphoinositide dependent protein kinase-1	−1.24		
SLC2A4	Solute carrier family 2 member 4 (GLUT4)	−1.62	Yes	
VAMP2	Vesicle-associated membrane protein 2	1.28		
EHD1	EH-domain containing 1	−1.29		
SNX26	Sorting nexin 26	−1.33		
SORBS1	Sorbin and SH3 domain containing 1	−1.30		
CBLC	Cas-Br-M ecotropic retroviral transf. sequence c	−1.29		
RAPGEF1	Rap guanine nucleotide exchange factor (GEF) 1	−1.24		
FOXO3A	Forkhead box O3	−1.50	Yes	Yes
SRF	Serum response factor	−1.28		
RHEB	Ras homolog enriched in brain	1.33	Yes	Yes
EIF4E	Eukaryotic translation initiation factor 4E	1.24		
RAF1	V-raf-1 murine leukemia viral oncogene homol. 1	1.29		
MAPK4	Mitogen-activated protein kinase 4	−1.31		
MAPK8	Mitogen-activated protein kinase 8	−1.23		
MAPK12	Mitogen-activated protein kinase 12	−1.26		
MAP2K7	Mitogen-activated protein kinase kinase 7	−1.53		
MAP4K4	Mitogen-act. protein kinase kinase kinase kinase 4	−1.44		
MINK	Misshapen-like kinase 1	−1.32		
*Modulators of Insulin Action*				
PTPN11	Protein tyrosine phosphatase, non-R type 11	−1.37		
MAPK8	Mitogen-activated protein kinase 8	−1.23		
SOCS3	Suppressor of cytokine signaling 3	−1.36		
IKBKB	Inhibitor of κ light polypept. gene enhancer in B-cells, kinase β	−1.32		
PRKCA	Protein kinase C, alpha	−1.36		
PRKCQ	Protein kinase C, theta	−1.3		
PPP1CB	Protein phosphatase 1, catalytic subunit, β isoform	−1.34		
PPM1A	Protein phosphatase 1A, Mg-dependent, α isoform	−1.22		
PPM1B	Protein phosphatase 1B, Mg-dependent, β isoform	−1.22		
PPP1R9B	Protein phosphatase 1, regulatory subunit 9B	−1.27		
PPP2CB	Protein phosphatase 2, catalytic subunit, β isoform	−1.21		
PPP2R5B	Protein phosphatase 2, regulatory subunit B', β	−1.25		
*Metabolic Regulation*				
PFKL	Phosphofructokinase, liver	−1.34		
LIPE	Lipase, hormone-sensitive	−1.34		
GYS1	Glycogen synthase 1	−1.29		
HK2	Hexokinase 2	−2.75	Yes	Yes
LPL	Lipoprotein lipase	−1.86	Yes	Yes
PHKA1	Phosphorylase kinase, alpha 1	−1.51	Yes	Yes
LDHB	Lactate dehydrogenase B	−1.90	Yes	Yes
*Mitochondrial Function*				
NDUFS1	NADH dehydrogenase 1	−1.60	Yes	Yes
NDUFS2	NADH dehydrogenase 2	−1.37		
HK2	Hexokinase 2	−2.75	Yes	Yes
NNT	Nicotinamide nucleotide transhydrogenase	−1.55		Yes
MTRR	Methyltransferase reductase	−1.45		Yes
POLG	Polymerase gamme	−1.50		Yes
PGC1α	PPARγ coactivator 1α	−1.05	Yes *	
PGC1β	PPARγ coactivator 1β	1.18	Yes	
*Collagens*				
COL1A1	Collagen, type I, alpha 1	−1.30	Yes	
COL3A1	Collagen, type III, alpha 1	−1.43	Yes	
*Miscellaneous*				
HDAC7A	Histone deacetylase 7A	−1.54	Yes	Yes
TCF7L2	Transcription factor 7-like 2	1.11	Yes	
KIF1B	Kinesin family member 1B	−1.23	Yes	
GDF8	Growth differentiation factor 8	1.56		

Table listing differentially expressed genes in people with type 2 diabetes mentioned in the results and discussion section grouped according to function/pathway classification. The fold changes (FC) are listed for each gene. It is also indicated whether or not the microarray result has been confirmed with qRT-PCR and whether or not the result applies to all dChip criteria used. The asterisk (*) indicate that *PGC1α* was found to be slightly down-regulated in the qRT-PCR experiment, which was not the case in the microarray experiment.

**Table 3:** Genes differentially expressed in first degree relatives of people with type 2 diabetes.

Gene symbol	Gene name	FC microarray	qRT-PCR	DChip criteria
*Insulin Signaling*				
GAB1	GRB2-associated binding protein 1	1.25	Yes	
PIK3CA	Phosphoinositide-3-kinase, catalytic, alpha	1.21	Yes	
PIK3CB	Phosphoinositide-3-kinase, catalytic, beta	1.25		
PIK3R3	Phosphoinositide-3-kinase, regulatory subunit 1	−1.22		
PIK3C3	Phosphoinositide-3-kinase, class 3	1.21		
TBC1D4	TBC1 domain family, member 4	1.24		
SORBS1	Sorbin and SH3 domain containing 1	1.27		
RHOQ	Ras homolog gene family, member Q	1.27		
SLC2A4	Solute carrier family 2 member 4 (GLUT4)	−1.04	Yes	
FOXO3A	Forkhead box O3	1.22	Yes	
RPS6KB1	Ribosomal protein S6 kinase, 70kDa, polypept. 1	1.30		
SOS2	Son of sevenless homolog 2	1.25		
MAPK8	Mitogen-activated protein kinase 8	1.23		
MAP3K2	Mitogen-activated protein kinase kinase kinase 2	1.23		
MAP3K7	Mitogen-activated protein kinase kinase kinase 7	1.21		
MAP4K3	Mitogen-act protein kinase kinase kinase kinase 3	1.24		
RPS6KA3	Ribosomal protein S6 kinase, 90kDa, polypeptide 3	1.21		
*Modulators of Insulin Action*				
PTPN11	Protein tyrosine phosphatase, non-R type 11	1.23		
MAPK8	Mitogen-activated protein kinase 8	1.23		
PRKAA2	Protein kinase, AMP-act., alpha 2 catalytic subunit	1.22		
*Metabolic Regulation*				
HK2	Hexokinase 2	−1.42	Yes	
LDHB	Lactate dehydrogenase B	−1.62	Yes	Yes
LPL	Lipoprotein lipase	−1.31	Yes	
*Mitochondrial Function*				
PGC1α	PPARγ coactivator 1α	1.05	Yes	
PGC1β	PPARγ coactivator 1β	−1.18	Yes	
HK2	Hekokinase 2	−1.42	Yes	
*Collagens*				
COL1A1	Collagen, type I, alpha 1	−1.57	Yes	Yes
COL3A1	Collagen, type III, alpha 1	−1.53	Yes	yes
*Miscellaneous*				
TCF7L2	Transcription factor 7-like 2	−1.15	Yes	
KIF1B	Kinesin family member 1B	1.51	Yes	Yes
GDF8	Growth differentiation factor 8	1.76	Yes	Yes
PDLIM5	PDZ and LIM domain 5	1.63	Yes	yes
TncRNA	Trophoblast-derived noncoding RNA	1.67		Yes
GOLGA8A	Golgi autoantigen, golgin subfamily a, 8A	1.57		Yes
ARID5B	AT rich interactive domain 5B	1.43		Yes
LONRF2	LON peptidase NT domain & ring finger 2	1.46		Yes

Table listing differentially expressed genes in first degree relatives mentioned in the results and discussion section grouped according to function/pathway classification. The fold changes (FC) are listed for each gene. It is also indicated whether or not the microarray result has been confirmed with qRT-PCR and whether or not the result applies to all dChip criteria used.

Using the same cutoffs, 11 genes were found to be differentially expressed in the ‘R’ group compared to controls, however this comparison had a FDR >5% (Figure 1). The 11 genes were the following: Collagen 1 alpha 1 (*COL1A1*), collagen 3 alpha 1 (*COL3A1*), growth differentiation factor 8 (*GDF8*), kinesin family member 1B (*KIF1B*), lactate dyhydrogenase B (*LDHB*), PDZ and LIM domain 5 (*PDLIM5*), trophoblast-derived noncoding RNA (*TncRNA*), golgi autoantigen, golgin subfamily A 8A (*GOLGA8A*), AT rich interactive domain 5B (*ARID5B*), LON peptidase N-terminal domain and ring finger 2 (*LONRF2*), and an EST (Table 3). Due to the higher FDR for this comparison, changes in the majority of these genes were validated by qRTPCR (Table 3 and online Figure S1).

Two genes, *LDHB* and *TncRNA*, were found to be differentially expressed in both the ‘D’ and the ‘R’ group. The function of TncRNA is currently unknown whereas LDHB is a key-enzyme in anaerobic glycolysis. *LDHB* was found to be downregulated in both the ‘R’ and the ‘D’ group compared to controls.

Generally, the majority of fold changes were found to be modest (between 1.2 and 1.4) with some exceptions like *HK2*, which was downregulated 2.75 times in the ‘D’ group compared to controls.

### Quantitative RT-PCR results

Thirteen genes found to be significantly differentially expressed in the ‘D’ group according to the chosen cutoffs in dChip, were further investigated by qRT-PCR (online Figure S1A). All genes were found to be regulated in the same direction with both methods, however two of the genes (*IRS2* and *RHEB* (Ras homolog enriched in brain)) did not live up to the FC>1.2 criteria.

Six of the 11 genes differentially expressed in the ‘R’ group were investigated by qRT-PCR (online Figure S1B). All genes were found to be regulated in the same direction with either method, however two genes (*COL1A1* and *COL3A1*) did not meet the FC>1.2 criteria (−1.18, and −1.19 respectively).

Additionally, genes of particular interest not found on the dChip derived genelists where investigated with qRT-PCR and the results were compared to microarray results (online Figure S1C). Using the qRT-PCR approach it was found that *PGC1α* (PPARγ coactivator 1α) was slightly downregulated in the ‘D’ group (FC = −1.20), and *PGC1β* (PPARγ coactivator 1 β) was downregulated in the ‘R’ group (FC = −1.35). However, these differences were not statistically significant.

All Ct averages, standard deviations, and fold changes can be seen in the online Table S2.

### Functional analysis using GenMAPP/MAPPFinder and Ingenuity Pathway Analysis

The data were compared between groups by functional analyses using GenMAPP/MAPPFinder. Fold changes and p-values calculated in dChip were imported to the program, and the cutoffs were the following: FC>1.2, p-value <0.05, and the mean expression value >30. A higher number of genes applied to these criteria, than in the dChip analyses, where additional cutoffs were present.

The top three functions/pathways for each comparison are shown in Table 4. It is striking that the most significantly upregulated pathway in the first degree relatives is Insulin Signaling, whereas it is the single most downregulated pathway in people with type 2 diabetes. These results are highly significant even when adjusting for multiple testing in MAPPFinder (Adjusted p-value). The only other significant pathway after adjusting for multiple testing is MAPK signaling, which is downregulated in the ‘D’ group. Generally, the majority of pathways/functions were upregulated in the relatives and downregulated in people with type 2 diabetes. It was also a general tendency that several pathways upregulated in the ‘R’ group become downregulated in the ‘D’ group. Besides insulin signaling, this was for example the case for genes involved in glycogen metabolism, muscle development, and apoptosis. Genes involved in protein synthesis were overall upregulated in both groups. Genes involved in mitochondrial function and electron transport were generally downregulated, however these functions were not found to be significantly changed compared to controls. Another notion was that several serine/threonine phosphatases had a decreased expression in skeletal muscle from the ‘D’ group compared to controls (*PPM1A*, *PPM1B*, *PPP1R9B*, *PPP2CB*, and *PPP2R5B*).

**Table 4:** Pathways/functions regulated on gene level.

Pathway/function	Z-score	Permuted p-value	Adjusted p-value	
*First degree relatives*				
**Insulin signaling**	**7.06**	**<0.001**	**0.005**	Upregulated
TGF-β signaling	6.25	<0.001	0.068	Upregulated
RNA splicing	5.76	<0.001	0.089	Upregulated
Focal adhesion	6.52	<0.001	0.140	Downregulated
Inorganic anion transport	4.18	0.002	0.740	Downregulated
Inflammatory response pathway	5.59	0.003	0.326	Downregulated
*People with type 2 diabetes*				
Apoptosis	4.31	<0.001	0.388	Upregulated
Protein modification	2.88	0.005	0.979	Upregulated
Cell cycle G1 to S control reactome	3.47	0.006	0.676	Upregulated
**Insulin signaling**	**6.17**	**<0.001**	**0.002**	Downregulated
**MAPK signaling**	**5.78**	**<0.001**	**0.002**	Downregulated
G-protein signaling	4.53	<0.001	0.078	Downregulated

The three most significantly upregulated and the three most significantly downregulated pathways/functions in skeletal muscle from people with type 2 diabetes and first degree relatives. Results were obtained employing the program GenMAPP/MAPPFinder. Criteria were set as described in the Methods section. Pathways found to be significantly altered after correction for multiple testing (adjusted p-value) are depicted in bold writing. Interestingly, the insulin signaling pathway was the highest ranked upregulated pathway in the first degree relative group, whereas it was found to be the top ranked downregulated pathway in people with type 2 diabetes.

Functional analyses were also made using the Ingenuity Pathway Analysis (IPA) program, looking at general regulation of signaling pathways not discriminating between up- and downregulation of specific genes. The same cutoffs were used as in the GenMAPP/MAPPFinder analyses. Overall, IPA analyses confirmed the result obtained from the GenMAPP/MAPPFinder analysis; namely that insulin signaling is the main signaling pathway altered in both groups (data not shown).

**Figure 2:**
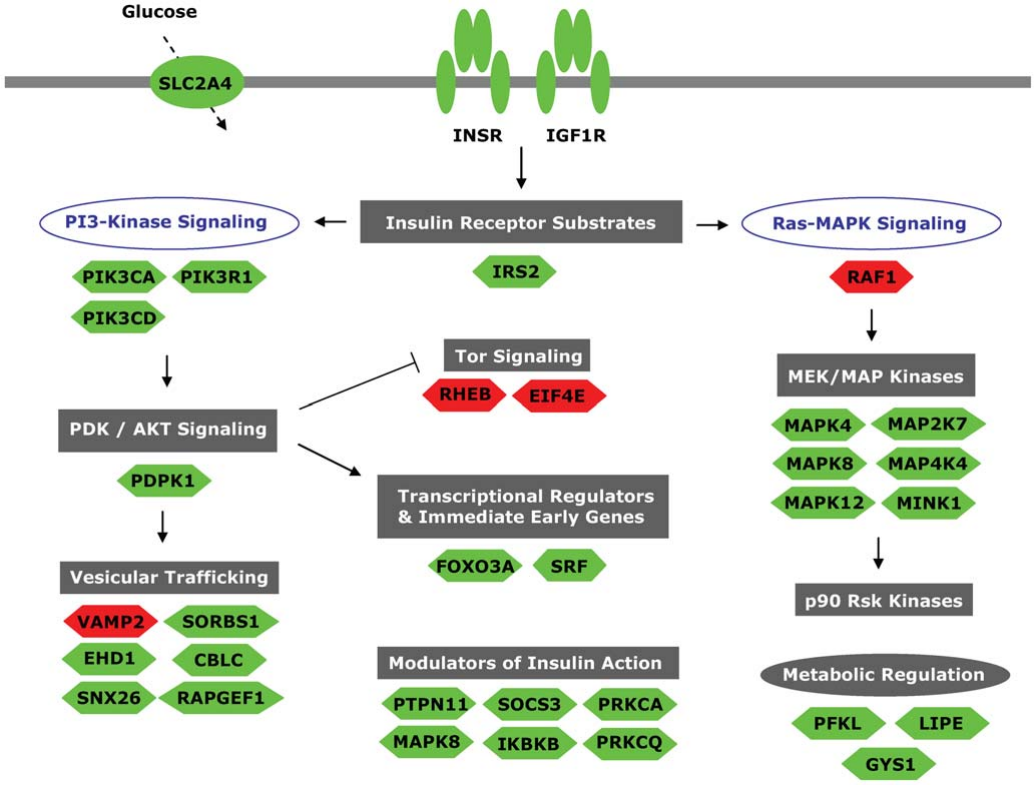
Regulation of insulin signaling in people with type 2 diabetes. The insulin signaling pathways were found to be significantly downregulated on the gene expression level using the program GenMAPP/MAPPFinder. Analysis criteria were set as described in the Methods section. Underneath each section of the pathway, genes found to have an increased expression are depicted in red, and genes found to have a decreased expression are depicted in green. Figure adapted from GenMAPP.

**Figure 3:**
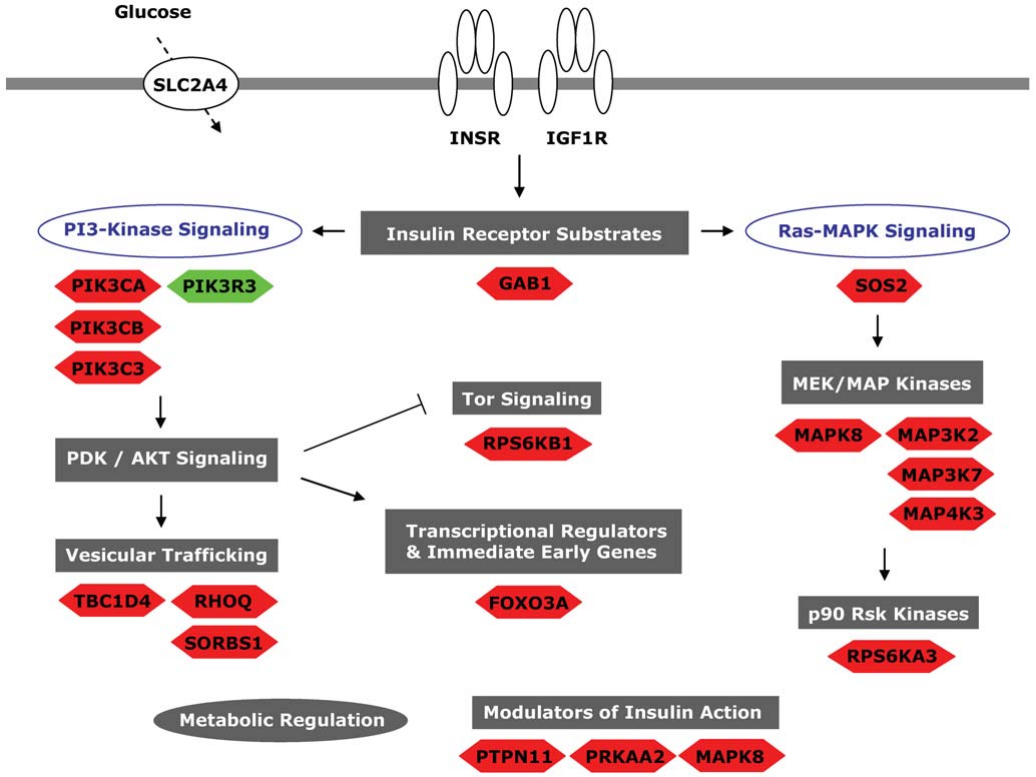
Regulation of insulin signaling in first degree relatives of people with type 2 diabetes. The insulin signaling pathways were found to be significantly upregulated on the gene expression level using the program GenMAPP/MAPPFinder. Analysis criteria were set as described in the Methods section. Underneath each section of the pathway, genes found to have an increased expression are depicted in red, and genes found to have a decreased expression are depicted in green. Figure adapted from GenMAPP.

The alterations in expression of genes involved in insulin signaling found in the GenMAPP/MAPPFinder analyses can be seen in Figure 2 and Figure 3. Some of the affected genes are overlapping but the majority varies between the ‘D’ and the ‘R’ group. Several of the genes in this analysis did not live up to all criteria set in the dChip analysis. The FCs observed for a subset of the genes have been confirmed with additional qRT-PCR results (Table 2 and Table 3)

### Protein expression of the Insulin Receptor (IR) and PGC1α

**Figure 4:**
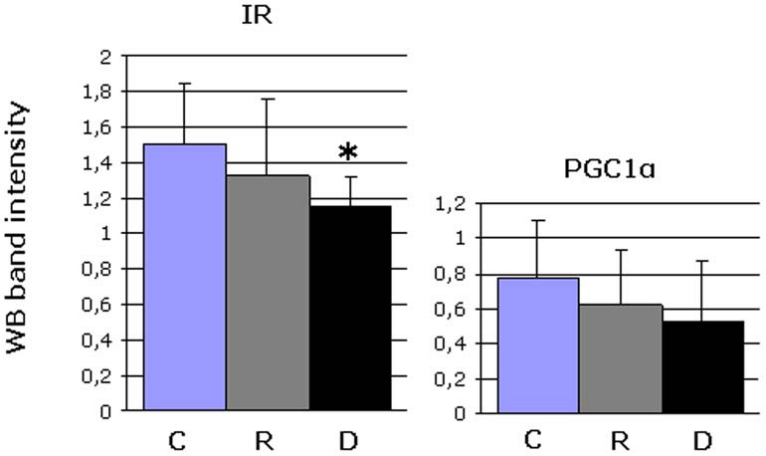
Protein expression of IR and PGC1α. Western blot (WB) analyses were performed for the IR and PGC1α for all samples used in the microarray study in order to verify mRNA results on protein level. Band intensities were determined and the average result for each group is shown in this figure. The only significant difference between groups was found to be a downregulation of the IR in the diabetic group compared to controls (unpaired t-test, p-value <0.05). This result fits with what was observed in the microarray study.

In order to validate our results at the gene expression level, protein levels of the IR and PGC1α were determined by western blot analysis for all samples of the 3 experimental groups. Figure 4 shows the average band intensities for each group. The only significant difference found between groups was for the IR, which is downregulated in the ‘D’ group compared to controls. These results correspond with our findings at the gene level. For PGC1α it is striking that huge interpersonal variation exists in all three groups, and it is likely that this transcription factor is indeed downregulated in some relatives and type 2 diabetic patients, but not in all.

## Discussion

In this study, skeletal muscle biopsies from male subjects with type 2 diabetes, first degree relatives, and healthy controls were investigated at the gene expression level using the microarray technology. The first degree relatives were slightly hyperinsulinemic in the fasting state and only mildly insulin resistant compared to type 2 diabetics, making them as close to the background population as possible. The same level of insulin resistance has previously been found in first degree relatives [21]. The elevated fasting plasma insulin levels in the first degree relatives support the notion that they are in the pre-diabetic stage probably on their way to develop overt insulin resistance. The patients with type 2 diabetes were obese, and as expected they had elevated fasting glucose and plasma FFA levels compared to both controls and first degree relatives (Table 1).

The biopsies were taken after a 2 hour hyperinsulinemic euglycemic clamp thereby ensuring a constant and controlled metabolic environment. When analyzing the data it should be kept in mind that for genes regulated by insulin, any change in expression could simply be a direct consequence of insulin resistance, since the muscle tissue is subjected to high levels of insulin during the clamp. Nonetheless, all differences seen between groups are genuine differences since all groups were treated in the same way. Furthermore, we were unable to completely match subjects for advanced age and elevated BMI in this study, which are known characteristics of patients with overt type 2 diabetes. Accordingly, we cannot exclude the possibility that age and/or BMI per se contributed to the differences found in patients with type 2 diabetes. However, this does not change the overall finding and conclusion that genes involved in insulin signaling are upregulated in people at risk of – and prior to - type 2 diabetes development, and subsequently are downregulated in the diabetic state.

Overall, differences in expression were found to be modest with FCs ranging between 1.2 and 1.4 for most genes. However, even small changes in gene expression can have a major biological impact, and using pathway analysis tools we show that even small changes on an individual gene level can lead to highly significant changes when combined for an entire pathway.

None of the genes that have been linked to increased risk of type 2 diabetes development in GWA studies [8], [9], [10] were found to have an altered expression in either group compared to the controls. This was validated by qRTPCR analysis for the gene *TCF7L2* (Table 2 and 3), in which SNPs so far have shown the strongest link to increased risk of type 2 diabetes. Most speculatively, it seems logical that changes in a transcription factor like TCF7L2 will lead to altered expression of other genes and not *TCF7L2* itself. However, it still needs to be verified that the SNPs associated to type 2 diabetes actually play a role in diabetes development and are not simply genetic markers for the disease.

Another general tendency was that genes and pathways found to be upregulated in the first degree relatives of type 2 diabetics were downregulated at the type 2 diabetic state. This phenomenon was found to be highly significant for the insulin signaling pathway.

### Expression of insulin signaling molecules is upregulated in first degree relatives and downregulated in subjects with type 2 diabetes

The most striking finding in this study was the highly significant increase in expression of genes involved in insulin signaling in skeletal muscle from first degree relatives of type 2 diabetics, and the significant downregulation of the same pathway in type 2 diabetic skeletal muscle samples (Table 4, Figure 2, and Figure 3). We hypothesize that the upregulation of the insulin signaling pathway at the gene expression level observed in the relatives could be an effective compensation for otherwise reduced insulin signaling activity. Since the first degree relatives are hyperinsulinemic they are most likely insulin resistant in a strictly molecular sense although not physiologically. Increased expression of insulin signaling molecules could possibly work in concert with increased levels of insulin protecting these individuals from insulin resistance and metabolic dysregulation. This compensation is later lost in type 2 diabetic muscle, and the insulin signaling pathways are at that state downregulated. Possible explanations for the loss of this compensatory mechanism in overt type 2 diabetes include glucose toxicity due to elevated plasma glucose levels, lipotoxicity due to elevated FFA levels, and/or failure of β-cell function. However, this remains speculative until specifically addressed in future studies.

Most of the genes affected in the ‘R’ and the ‘D’ group are not overlapping. This is for example the case for *SLC2A4* (*GLUT4* (Glucose transporter 4)), which is downregulated in the ‘D’ group and unaltered in the ‘R’ group. This observation can be explained by the fact that *SLC2A4* expression is increased during a hyperinsulinemic clamp in healthy muscle but not in type 2 diabetic muscle [22]. One of the few genes involved in insulin signaling found in this study to be upregulated in the type 2 diabetic muscle is *VAMP2* (Figure 2). This gene encodes a protein residing on the GLUT4 vesicle surface and plays an important role in the interaction between the vesicle and the plasma membrane target [23]. An increase in the expression of proteins promoting efficient GLUT4 trafficking and fusion to the membrane (like VAMP2) could be a way to compensate for a decreased amount of GLUT4 protein.

Insulin signaling defects observed in muscle from people with type 2 diabetes has previously been reported to be specific for the metabolism regulating part of the pathway, thereby leaving the MAP kinase part of the pathway intact [24]. However, we found that several of the MAP kinases were downregulated at the gene expression level (Figure 2). The decreased amount of MAP kinase expression could lead to a decreased serine/threonine phosphorylation of for example the IRS proteins, ultimately increasing insulin signaling activity as part of a compensatory mechanism directed against insulin resistance.

We also found that several serine/threonine phosphatases had a decreased expression in diabetic muscle compared to controls (*PPM1A*, *PPM1B*, *PPP1R9B*, *PPP2CB*, and *PPP2R5B*) (Table 2). Possibly, this reduction in phosphatase expression will translate into an increased level of serine/threonine phosphorylation further worsening the intensity of insulin resistance in these patients.

### OXPHOS genes and PGC1α/PGC1β

Oxidative phosphorylation (OXPHOS), which has previously been shown to be downregulated in both prediabetic relatives and people with type 2 diabetes [13], [14], was not found to be significantly different in either group in this study. Several factors can partly explain this divergence in results. In the study of Patti et al., all subjects were Mexican-Americans, biopsies were taken at basal levels and from groups of mixed sexes. Additionally, HuGeneFL arrays from Affymetrix representing 7,129 sequences were used in that particular study [14]. In comparison, the arrays used in the current study had more than 50,000 probesets representing approximately 47,000 transcripts. That fact alone is likely to result in different findings when it comes to pathway and functional analyses. In the study of Mootha et al., samples were taken after a hyperinsulinemic-euglycemic clamp, all subjects were of Caucasian origin, and the groups consisted of only males as in the present study. However, the arrays used (HG-U133A arrays from Affymetrix) covered only about half of the transcripts found on the arrays used in the current study [13].

Even though the OXPHOS genes as a group were not significantly changed at the expression level in first degree relatives or in type 2 diabetic patients, several individual genes involved in mitochondrial function and energy derivation had a decreased level of expression. NADH dehydrogenase 1 (*NDUFS1*), NADP transhydrogenase (*NNT*), 5-methyltetrahydrofolate-homocysteine methyltransferase reductase (*MTRR*), polymerase gamma (*POLG*), NADH dehydrogenase 2 (*NDUFS2*) were among others found to be down-regulated in the ‘D’ group (Table 2).

In this study, we could not detect any significant downregulation of *PGC1α* or *PGC1β* in muscle biopsies from group ‘R’ or group ‘D’ (Table 2 and Table 3). A significant decreased expression of these genes in pre-diabetic relatives and people with type 2 diabetes has previously been reported, contradicting the present results [13], [14]. However, a study of Karlsson et al. recently found that the mRNA expression of PGC1α and PGC1β in normo-glycemic first degree relatives was within the same range as for healthy controls, which supports the findings of the current study [17]. To clarify this matter, we determined the protein expression of PGC1α in all three experimental groups, and found that PGC1α indeed looks like it is downregulated in some first degree relatives and diabetic patients, but not in others. Due to the high interpersonal variation the measured downregulation is not significant (Figure 4).

### Genes with altered expression levels in first degree relatives of type 2 diabetics

As previously mentioned, alterations in gene expression found in healthy first degree relatives of type 2 diabetics are good candidates when searching for underlying causes of the disease.

8 of the 11 genes found to be differentially expressed in muscle samples from first degree relatives had an increased level of expression compared to the controls. These genes include among others *KIF1B* and *GDF8*. Interestingly, the expression of *KIF1B* was downregulated in the ‘D’ group using both the microarray and the qRT-PCR approach. Both of these genes could turn out to play a crucial role in type 2 diabetes pathogenesis.

KIF1B has been shown to be highly involved in the transport of mitochondria and *KIF1B* heterozygous mice have an impaired transport of synaptic vesicle precursors and suffer from a high degree of muscle weakness [25], [26]. Type 2 diabetes has been associated with a decreased mitochondrial level in skeletal muscle [27]. An upregulation of mitochondrial transport by upregulation of *KIF1B* could possibly be a way to compensate for a supposed decreased mitochondrial level. Interestingly, one of the gene regions recently found to associate with type 2 diabetes contains *KIF11* – another kinesin family member [8].

GDF8 is also known as myostatin, which works as an inhibitor of skeletal muscle growth and is a member of the TGF-beta family. Myostatin has been suggested as a good candidate for therapeutic intervention in diseases with loss of muscle mass, including diabetes. Indeed, an increased expression of this gene has been reported in skeletal muscle from chronic muscle wasting conditions such as cachexia and aging in human and animal models [28]–[30]. Finding *GDF8* (myostatin) to be upregulated in healthy first degree relatives in this study suggests that this factor could play an initiating role in the muscle wasting observed in many diabetic patients and potentially in the development of insulin resistance in the prediabetic stage.

The only gene with a know function found to have altered expression levels in both the first degree relatives and the type 2 diabetics was *LDHB*. LDHB catalyzes the conversion of pyruvate to lactate in the anaerobic glycolytic process and is therefore crucial for normal energy homeostasis. Mitochondrial ATP synthesis has been reported to be down in insulin resistant but non-diabetic offspring of parents with type 2 diabetes as well as in type 2 diabetic patients [31]–[32]. The results of this study suggest that it is not only mitochondrial ATP production that is impaired in these individuals but also ATP generation via the anaerobic pathway. Since mitochondrial oxidative phosphorylation and LDHB in a way compete for same pool of pyruvate it is also a possibility that decreased levels of LDHB is a compensatory mechanism in response to impaired mitochondrial function as more pyruvate will be available for acetyl Coenzyme A conversion.

### Additional genes with altered gene expression levels in type 2 diabetic skeletal muscle

Several interesting genes were found to be differentially expressed in the ‘D’ group compared to controls using the dChip program. One of the genes with the largest FCs is *HK2* (FC = −2.75, Table 2). This gene has previously been shown to have an impaired expression in type 2 diabetic skeletal muscle [33]. Furthermore, it has been shown that *HK2* expression is stimulated by insulin in healthy individuals but not in obese or type 2 diabetes patients [34]. This can explain the decrease in expression of *HK2* in the type 2 diabetics since subjects were submitted to a hyperinsulinemic clamp before samples were taken.

*HDAC7A* (histone deacetylase 7A) was also found to have a reduced expression in muscle from type 2 diabetes patients (FC = −1.54, Table 2). It has previously been hypothesized that an abnormal acetylation/deacetylation pattern and thereby an altered regulation of gene expression could play a role in the pathogenesis of type 2 diabetes [35].

In summary, this study for the first time shows a striking difference in the gene expression of insulin signaling molecules between people with type 2 diabetes and first degree relatives in skeletal muscle. Insulin signaling was significantly upregulated in first degree relatives, and significantly downregulated in type 2 diabetes patients. We suggest that increased expression of insulin signaling molecules work in concert with increased levels of insulin protecting people in the pre-diabetic state from insulin resistance and metabolic dys-regulation. However, future studies are needed to clarify the molecular basis and clinical importance of this phenomenon, and it will be interesting to see if the same results will be obtained in other tissues like pancreatic islets and adipose tissue.

Furthermore, several potentially important genes regarding the underlying causes of insulin resistance and type 2 diabetes (for example *KIF1B* and *GDF8*) have been identified and shown to have different gene expression levels in healthy first degree relatives compared to controls. These new findings in first degree relatives could potentially be used as a diagnostic tool in the prediction of type 2 diabetes. Further investigations in the future will be imperative in clarifying specific possible roles of these results in type 2 diabetes pathogenesis.

## Supporting Information

Figure S1 Validation of selected genes found to be differentially expressed in skeletal muscle compared to healthy control samples in a dChip analysis of the microarray results. Fold changes obtained in the microarray study are compared to fold changes obtained using qRT-PCR.The grey stabled line indicates the 1.2 cutoff. The qRT-PCR results are averages of two individual experiments employing TagMan Low Density Arrays from Applied Biosystems. A: People with type 2 diabetes. All genes were found to be regulated in the same direction with both methods. Two genes, RHEB and IRS2, did not live up to the FC > 1.2 criteria using the qRT-PCR approach. B: First degree relatives. All genes were found to be regulated in the same direction with both methods. Two genes, COL1A1 and COL3A1, did not live up to the FC > 1.2 criteria using the qRT-PCR approach. C: Genes investigated by qRT-PCR that did not live up to all criteria in the dChip analysis. The fold changes found for type 2 diabetic muscle compared to controls are on the left side of the figure, and fold changes found for skeletal muscle from first degree relatives are depicted in the right part of the figure. For standard deviations for qRT-PCR results please refer to Table S2.
(0.10 MB DOC)

Table S1 Table showing all genes found to apply to all criteria set in a dChip analysis comparing people with type 2 diabetes with controls, and first degree relatives with controls. Fold changes (FC) and gene names are listed.
(0.22 MB DOC)

Table S2 Averages of biological replicate Ct values and their standard deviation (SD). Ct values for 29 genes were determined for all samples. The first degree relative and control groups consisted of 15 people and each sample was run in two independent experiments. The type 2 diabetes group consisted of 5 people, and each sample was run in three independent experiments. ΔCt values (normalization using endogenous control value - in this case PPIA) and averages were calculated. Relative fold changes were calculated as: FC = 2-ΔΔCt.
(0.09 MB DOC)
