# Supplementary figures and images for: Gene Expression in Skeletal Muscle Biopsies from People with Type 2 Diabetes and Relatives: Differential Regulation of Insulin Signaling Pathways
Source: PLoS One. 2009 Aug 11;4(8):e6575. doi: 10.1371/journal.pone.0006575 (PMC2719801; doi:10.1371/journal.pone.0006575)

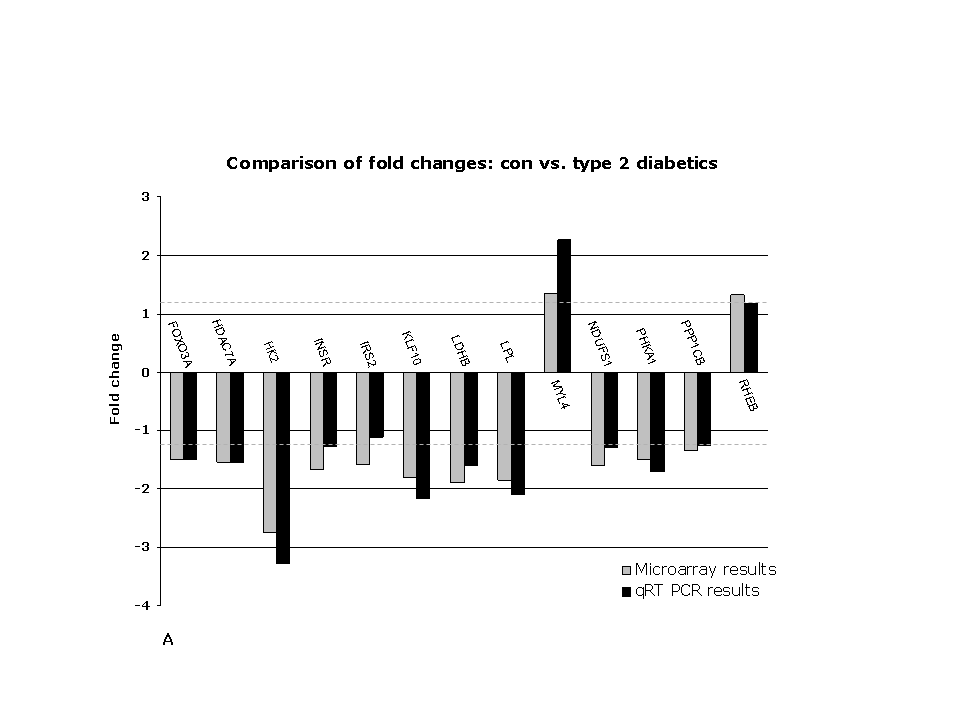


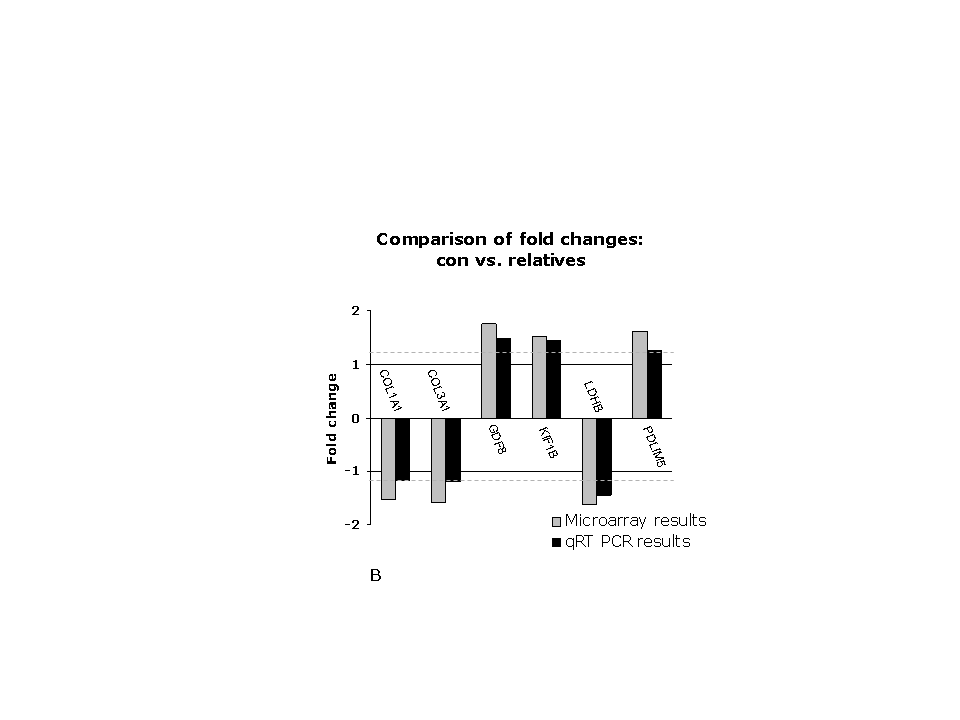


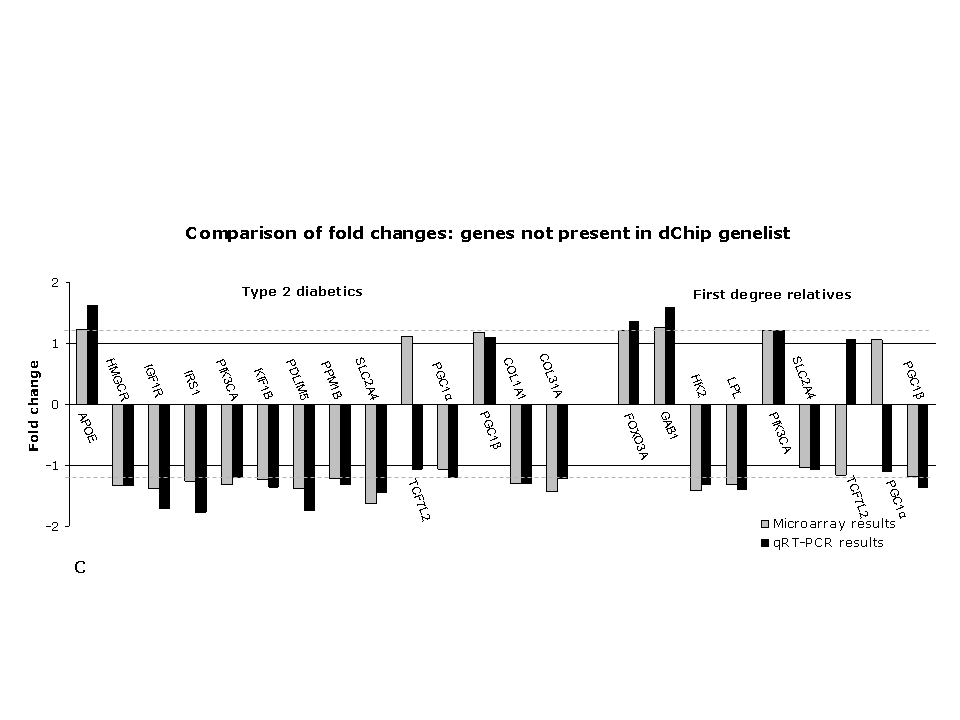

Supplement: Figure S1 — Validation of selected genes found to be differentially expressed in skeletal muscle compared to healthy control samples in a dChip analysis of the microarray results. Fold changes obtained in the microarray study are compared to fold changes obtained using qRT-PCR.The grey stabled line indicates the 1.2 cutoff. The qRT-PCR results are averages of two individual experiments employing TagMan Low Density Arrays from Applied Biosystems. A: People with type 2 diabetes. All genes were found to be regulated in the same direction with both methods. Two genes, RHEB and IRS2, did not live up to the FC > 1.2 criteria using the qRT-PCR approach. B: First degree relatives. All genes were found to be regulated in the same direction with both methods. Two genes, COL1A1 and COL3A1, did not live up to the FC > 1.2 criteria using the qRT-PCR approach. C: Genes investigated by qRT-PCR that did not live up to all criteria in the dChip analysis. The fold changes found for type 2 diabetic muscle compared to controls are on the left side of the figure, and fold changes found for skeletal muscle from first degree relatives are depicted in the right part of the figure. For standard deviations for qRT-PCR results please refer to Table S2. (0.10 MB DOC) [file pone.0006575.s001.doc]
