# Supplementary material for: Gene Expression in Skeletal Muscle Biopsies from People with Type 2 Diabetes and Relatives: Differential Regulation of Insulin Signaling Pathways
Source: PLoS One. 2009 Aug 11;4(8):e6575. doi: 10.1371/journal.pone.0006575 (PMC2719801; doi:10.1371/journal.pone.0006575)
Supplement: Table S1 — Table showing all genes found to apply to all criteria set in a dChip analysis comparing people with type 2 diabetes with controls, and first degree relatives with controls. Fold changes (FC) and gene names are listed. (0.22 MB DOC) [file pone.0006575.s002.doc]

| **Table S1**  Fold changes for all genes (probesets) found to be significant in dChip analyses | | | |
| --- | --- | --- | --- |
|  |  |  |  |
| **Gene Symbol** | **Gene name** | **C versus D FC** | **C versus R FC** |
| BRSK1 | BR serine/threonine kinase 1 | -1.48 |  |
| KLHL34 | kelch-like 34 (Drosophila) | -2.37 |  |
| ST3GAL3 | ST3 beta-galactoside alpha-2,3-sialyltransferase 3 | 1.35 |  |
| RGS5 | regulator of G-protein signalling 5 | -2.03 / -1.68 |  |
| TTN | Titin | -2.1 / -1.88 |  |
| LDHB | lactate dehydrogenase B | -1.9 / -1.88 | -1.62 / -1.65 |
| HNRPA0 | heterogeneous nuclear ribonucleoprotein A0 | -1.36 |  |
| HNRPF | heterogeneous nuclear ribonucleoprotein F | -1.34 |  |
| PPP1CB | protein phosphatase 1, catalytic subunit, beta isoform | -1.34 |  |
| GOLGA4 | golgi autoantigen, golgin subfamily a, 4 | -1.47 |  |
| GSPT1 | G1 to S phase transition 1 | -1.44 |  |
| KLF10 | Kruppel-like factor 10 | -1.81 |  |
| RXRA | retinoid X receptor, alpha | -1.38 |  |
| FBN1 | fibrillin 1 | -1.53 |  |
| NNT | nicotinamide nucleotide transhydrogenase | -1.55 |  |
| DDIT4 | DNA-damage-inducible transcript 4 | 1.66 |  |
| LSM5 | LSM5 homolog, U6 small nuclear RNA associated | 1.84 |  |
| S100A8 | S100 calcium binding protein A8 | -2.57 |  |
| HK2 | Hexokinase 2 | -2.75 |  |
| RAPGEF2 | Rap guanine nucleotide exchange factor (GEF) 2 | -1.65 |  |
| MTRR | 5-Mtetrahydrofolate-homocyst. Mtransferase reductase | -1.45 |  |
| HES1 | hairy and enhancer of split 1, (Drosophila) | -2.11 / -2.39 |  |
| CUL5 | cullin 5 | -1.46 |  |
| LPL | Lipoprotein lipase | -1.86 / -1.85 |  |
| PPFIBP1 | PTPRF interacting protein, binding protein 1 | 1.32 |  |
| FOXO3 | forkhead box O3 | -1.5 |  |
| ENPP4 | ectonucleotide pyrophosphatase/phosphodiesterase 4 | 1.43 |  |
| TBC1D8 | TBC1 domain family, member 8 | -1.55 |  |
| IARS | isoleucyl-tRNA synthetase | -1.31 |  |
| TAOK2 | TAO kinase 2 | -1.39 |  |
| GJA4 | Gap junction protein, alpha 4 | -1.38 |  |
| FABP7 | fatty acid binding protein 7, brain | -2.66 |  |
| MAPK8IP2 | mitogen-act. protein kinase 8 interacting protein 2 | -1.48 |  |
| SIP1 | survival of motor neuron protein interacting protein 1 | 1.81 |  |
| CENTB1 | centaurin, beta 1 | -1.53 |  |
| BARD1 | BRCA1 associated RING domain 1 | -1.5 |  |
| PFDN4 | prefoldin subunit 4 | 1.54 |  |
| PHKA1 | phosphorylase kinase, alpha 1 (muscle) | -1.51 / -1.58 |  |
| SETBP1 | SET binding protein 1 | -1.47 |  |
| PTAFR | platelet-activating factor receptor | -1.81 |  |
| ELAVL3 | ELAV-like 3 | -1.47 |  |
| GLRX | glutaredoxin (thioltransferase) | 1.57 / 1.59 |  |
| TCF15 | transcription factor 15 (basic helix-loop-helix) | -1.73 |  |
| GADD45B | growth arrest and DNA-damage-inducible, beta | -1.52 |  |
| CITED2 | Cbp/p300-interacting transactivator, with Glu/Asp-rich carboxy-terminal domain, 2 | 1.63 / 1.73 |  |
| MACF1 | microtubule-actin crosslinking factor 1 | -1.48 |  |
| GABARAPL1 | GABA(A) receptor-associated protein like 1 | -1.51 / -1.52 |  |
| CADM1 | Cell adhesion molecule 1 | -1.39 |  |
| IRS2 | Insulin receptor substrate 2 | -1.58 |  |
| SLC19A2 | solute carrier fam 19 (thiamine transp.) member 2 | -1.51 |  |
| MYL4 | myosin, light chain 4, alkali; atrial, embryonic | 1.35 / 1.32 |  |
| BBC3 | BCL2 binding component 3 | -1.46 |  |
| PDE4DIP | phosphodiesterase 4D interacting protein (myomegalin) | 2.04 |  |
| CDKN2C | cyclin-dependent kinase inhibitor 2C | -1.38 |  |
| DCN | decorin | -1.42 |  |
| DST | dystonin | -1.57 |  |
| TLN2 | Talin 2 | -1.34 |  |
| SAMD4A | sterile alpha motif domain containing 4A | -1.4 |  |
| RHEB | Ras homolog enriched in brain | 1.33 |  |
| FABP3 | fatty acid binding protein 3, muscle and heart | 1.5 |  |
| TncRNA | Trophoblast-derived noncoding RNA | -1.84 / -1.52 / -1.67 | 1.67 |
| ALPK3 | alpha-kinase 3 | -1.44 |  |
| NEB | Nebulin | -1.7 |  |
| SFTPC | surfactant, pulmonary-associated protein C | -1.58 |  |
| MAP2K3 | Mitogen-activated protein kinase kinase 3 | 1.4 |  |
| MCM3AP | minichromosome maintenance complex component 3 associated protein | 1.32 |  |
| RECK | reversion-inducing-cysteine-rich protein with kazal motifs | 1.44 |  |
| SYNJ2 | synaptojanin 2 | -1.59 |  |
| GPR144 | G protein-coupled receptor 144 | -1.51 |  |
| SOX15 | SRY (sex determining region Y)-box 15 | -1.52 |  |
| RPS10 | ribosomal protein S10 | 1.44 |  |
| POLG | polymerase (DNA directed), gamma | -1.5 |  |
| YWHAB | tyrosine 3-monooxygenase/tryptophan 5-monooxygenase activation protein, β | 1.38 |  |
| RPS26 | ribosomal protein S26 | 1.56 |  |
| SLMO2 | slowmo homolog 2 | 1.48 |  |
| HDAC7A | histone deacetylase 7A | -1.54 |  |
| MAP4K4 | mitogen-activated protein kinase kinase kinase kinase 4 | -1.44 |  |
| SLC38A1 | solute carrier family 38, member 1 | -2.16 |  |
| FHOD3 | formin homology 2 domain containing 3 | -1.51 |  |
| FBXO40 | F-box protein 40 | -1.46 |  |
| USP25 | ubiquitin specific peptidase 25 | -1.49 |  |
| DNAH3 | Dynein, axonemal, heavy chain 3 | 1.49 |  |
| APOL2 | apolipoprotein L, 2 | -1.44 |  |
| USP47 | ubiquitin specific peptidase 47 | -1.33 / -1.43 |  |
| AKAP13 | A kinase (PRKA) anchor protein 13 | -1.5 |  |
| ZNF43 | zinc finger protein 43 | 1.41 |  |
| TMEM30A | transmembrane protein 30A | 1.42 |  |
| SAP30L | SAP30-like | -1.59 |  |
| RPP25 | ribonuclease P 25kDa subunit | -1.56 |  |
| PDE7A | phosphodiesterase 7A | -1.41 |  |
| TRIM4 | tripartite motif-containing 4 | 1.8 |  |
| HECTD1 | HECT domain containing 1 | -1.45 |  |
| FKBP5 | FK506 binding protein 5 | -1.49 |  |
| MRPS6 | mitochondrial ribosomal protein S6 | 1.4 |  |
| SPIRE1 | spire homolog 1 | -1.53 |  |
| ARRDC4 | arrestin domain containing 4 | -1.92 |  |
| HSPC159 | galectin-related protein | -1.47 |  |
| ADHFE1 | alcohol dehydrogenase, iron containing, 1 | -1.65 |  |
| MEGF8 | multiple EGF-like-domains 8 | -1.42 |  |
| INSR | Insulin receptor | -1.66 |  |
| ADAMTS10 | ADAM metallopeptidase w thrombospondin type 1 motif, 10 | -1.52 |  |
| KLF15 | Kruppel-like factor 15 | -1.61 |  |
| CAPS | calcyphosine | -1.36 |  |
| FBXO6 | F-box protein 6 | 1.5 |  |
| SCAND1 | SCAN domain containing 1 | 1.34 |  |
| PRO1268 | PRO1268 protein | 1.56 |  |
| NDUFS1 | NADH dehydrogenase (ubiquinone) Fe-S protein 1, 75kDa | -1.6 |  |
| TUSC5 | tumor suppressor candidate 5 | -1.55 |  |
| TFRC | Transferrin receptor (p90, CD71) | -1.55 |  |
| CENTD2 | centaurin, delta 2 | -1.5 |  |
| CPEB3 | cytoplasmic polyadenylation element binding protein 3 | -1.4 |  |
| HBEGF | heparin-binding EGF-like growth factor | -1.43 |  |
| COL3A1 | collagen, type III, alpha 1 |  | -1.53 |
| COL1A1 | collagen, type I, alpha 1 |  | -1.57 |
| GDF8 | growth differentiation factor 8 |  | 1.76 |
| GOLGA8A | golgi autoantigen, golgin subfamily a, 8A |  | 1.57 |
| KIF1B | kinesin family member 1B |  | 1.51 |
| ARID5B | AT rich interactive domain 5B (MRF1-like) |  | 1.43 |
| LONRF2 | LON peptidase N-terminal domain and ring finger 2 |  | 1.46 |
| PDLIM5 | PDZ and LIM domain 5 |  | 1.63 |
|  | CDNA FLJ26188 fis, clone ADG04821 |  | 1.41 |
|  | CDNA FLJ90128 fis, clone HEMBB1000276 | -1.83 / -2.01 |  |
| 1558048_x_at | gb:BG389789 | 1.72 |  |
| 210483_at | hypothetical protein MGC31957 | -1.54 |  |
| 213657_s_at | MRNA full length insert cDNA clone EUROIMAGE 375854 | -1.48 |  |
| 213979_s_at | Full-length cDNA clone CS0DF027YP13 | -1.63 |  |
| 214223_at | Transcribed locus, strongly similar to XP_001148966.1 hypothetical protein | -1.4 |  |
| LOC391132 | similar to 60S ribosomal protein L29 (P23) | 1.38 |  |
| MGC10997 | pseudogene MGC10997 | 1.41 |  |
| LOC643653 | similar to 60S ribosomal protein L35 | 1.42 |  |
| C20orf177 | chromosome 20 open reading frame 177 | 1.42 |  |
| 227762_at | Transcribed locus | -1.47 |  |
| 229116_at | CDNA clone IMAGE:30721737 | -2.23 |  |
| DKFZp434B1231 | eEF1A2 binding protein | -3.11 |  |
| 230503_at | Transcribed locus | -1.56 |  |
| 231161_x_at | Tripartite motif-containing 8 | -1.49 |  |
| LOC645895 | hypothetical LOC645895 | -1.5 |  |
| 232495_x_at | Clone 23900 mRNA sequence | 1.39 |  |
| 234675_x_at | CDNA: FLJ23566 fis, clone LNG10880 | 1.93 |  |
| LOC388692 LOC644634 | hypothetical gene supported by AK123662 hypothetical LOC644634 | -1.96 |  |
| LOC284454 | hypothetical protein LOC284454 | -1.35 |  |
| LOC643837 | hypothetical protein LOC643837 | -1.44 |  |
| 236576_at | Transcribed locus | -1.65 |  |
| 236657_at | Full length insert cDNA YI37C01 | 1.7 |  |
| LOC165186 | similar to RIKEN cDNA 4632412N22 gene | -1.84 |  |
| C16orf77 | chromosome 16 open reading frame 77 | -1.41 |  |
| 238267_s_at | Transcribed locus | -1.88 |  |
| LOC440836 | Similar to MGC52679 protein | -2.51 |  |
| 238351_x_at | Transcribed locus | -1.48 |  |
| FLJ45803 | FLJ45803 protein | 1.5 |  |
| 238767_at | Transcribed locus | -1.4 |  |
| 240854_x_at | gb:BF514007 | 1.38 |  |
| 241255_at | gb:BF433749 | -1.47 |  |
| 241617_x_at | gb:BE961949 | -2.25 |  |
| 241618_at | Transcribed locus | -1.61 |  |
| 242652_at | Erythrocyte membrane protein band 4.1-like 2 | -1.44 |  |
| 244156_at | Transcribed locus | -1.6 |  |
| 244447_at | Transcribed locus | -1.61 |  |
