# Supplementary material for: Gene Expression in Skeletal Muscle Biopsies from People with Type 2 Diabetes and Relatives: Differential Regulation of Insulin Signaling Pathways
Source: PLoS One. 2009 Aug 11;4(8):e6575. doi: 10.1371/journal.pone.0006575 (PMC2719801; doi:10.1371/journal.pone.0006575)
Supplement: Table S2 — Averages of biological replicate Ct values and their standard deviation (SD). Ct values for 29 genes were determined for all samples. The first degree relative and control groups consisted of 15 people and each sample was run in two independent experiments. The type 2 diabetes group consisted of 5 people, and each sample was run in three independent experiments. ΔCt values (normalization using endogenous control value - in this case PPIA) and averages were calculated. Relative fold changes were calculated as: FC = 2-ΔΔCt. (0.09 MB DOC) [file pone.0006575.s003.doc]

| **Table S2** Ct values, standard deviations, and fold changes | | | | | | | | |
| --- | --- | --- | --- | --- | --- | --- | --- | --- |
|  | **Controls** | | **People with type 2 diabetes** | | | **First degree relatives** | | |
| Gene | Average Ct | SD | Average Ct | SD | FC C vs. D | Average Ct | SD | FC C vs. R |
| APOE | 28.06502 | 1.603748 | 27.26907 | 0.500640 | 1.63 | 27.60084 | 0.227114 | 1.37 |
| COL1A1 | 26.45960 | 0.598018 | 26.80840 | 0.281480 | -1.30 | 26.68614 | 0.454324 | -1.18 |
| COL3A1 | 24.10784 | 0.721741 | 24.41219 | 0.256932 | -1.22 | 24.34872 | 0.459323 | -1.19 |
| FOXO3A | 26.13093 | 0.515535 | 26.58620 | 0.161259 | -1.50 | 25.68602 | 0.309137 | 1.35 |
| GAB1 | 28.52855 | 0.394175 | 28.39087 | 0.594194 | -1.04 | 27.84622 | 0.523404 | 1.59 |
| GDF8 | 26.67578 | 0.673472 | 26.76700 | 1.641811 | -1.16 | 26.08901 | 0.756886 | 1.49 |
| HDAC7A | 27.14353 | 0.379133 | 27.73995 | 0.585103 | -1.54 | 27.10611 | 0.262986 | 1.00 |
| HK2 | 26.84002 | 1.099827 | 28.34890 | 1.315311 | -3.28 | 27.21821 | 0.965388 | -1.31 |
| HMGCR | 28.03161 | 0.387031 | 28.34384 | 0.337968 | -1.33 | 28.02856 | 0.364322 | 1.00 |
| IGF1R | 27.13902 | 0.397438 | 27.75559 | 0.626175 | -1.70 | 27.03080 | 0.329960 | 1.07 |
| INSR | 25.49246 | 0.482019 | 25.64538 | 0.390743 | -1.28 | 25.31068 | 0.514009 | 1.12 |
| IRS1 | 25.81141 | 0.310999 | 26.38704 | 0.396597 | -1.77 | 25.79460 | 0.387420 | 1.00 |
| IRS2 | 27.25533 | 0.484963 | 27.26804 | 0.189383 | -1.12 | 27.09205 | 0.367884 | 1.11 |
| KIF1B | 27.67171 | 0.747908 | 28.03612 | 0.422392 | -1.35 | 27.12479 | 0.497761 | 1.45 |
| KLF10 | 23.35778 | 0.617366 | 24.63287 | 0.606654 | -2.16 | 23.47150 | 0.492623 | -1.09 |
| LDHB | 23.98978 | 0.939379 | 24.25611 | 0.350799 | -1.61 | 24.50535 | 0.781424 | -1.44 |
| LPL | 24.50127 | 0.862859 | 25.33494 | 0.316694 | -2.10 | 24.97430 | 0.683548 | -1.40 |
| MYL4 | 33.31996 | 1.034356 | 32.03582 | 0.980067 | 2.27 | 33.73264 | 0.804482 | -1.34 |
| NDUFS1 | 22.16073 | 0.327048 | 22.40086 | 0.310631 | -1.30 | 22.09088 | 0.323949 | 1.04 |
| PDLIM5 | 27.55762 | 0.487533 | 28.25766 | 0.427273 | -1.75 | 27.21966 | 0.688808 | 1.26 |
| PHKA1 | 23.34672 | 0.391316 | 24.11200 | 0.249176 | -1.71 | 23.19631 | 0.299314 | 1.10 |
| PIK3CA | 28.05388 | 0.344676 | 28.10134 | 0.214015 | -1.20 | 27.76584 | 0.294322 | 1.21 |
| PPARGC1A | 24.84768 | 0.551835 | 24.96466 | 0.237141 | -1.20 | 24.97190 | 0.711473 | -1.10 |
| PPARGC1B | 26.18109 | 0.437029 | 25.94287 | 0.114550 | 1.09 | 26.59992 | 0.725542 | -1.35 |
| PPM1B | 23.72818 | 0.428164 | 23.89513 | 0.126724 | -1.31 | 23.66698 | 0.422259 | 1.03 |
| PPP1CB | 21.32253 | 0.287576 | 21.48885 | 0.363619 | -1.25 | 21.20642 | 0.363653 | 1.07 |
| RHEB | 23.97029 | 0.443314 | 23.54641 | 0.278242 | 1.17 | 23.82779 | 0.405520 | 1.09 |
| SLC2A4 | 21.75614 | 0.424879 | 22.27713 | 0.389078 | -1.46 | 21.80944 | 0.366199 | -1.05 |
| TCF7L2 | 27.05703 | 0.354471 | 27.05438 | 0.255040 | -1.05 | 26.96819 | 0.290372 | 1.05 |
|  |  |  |  |  |  |  |  |  |
| PPIA | 23.89983 | 0.450684 | 23.74978 | 0.203939 |  | 23.88777 | 0.367084 |  |
|  |  |  |  |  |  |  |  |  |
